# n6-methyladenosine-modified circular RNA family with sequence similarity 126, member A affects cholesterol synthesis and malignant progression of prostate cancer cells by targeting microRNA-505-3p to mediate calnexin

**DOI:** 10.7150/jca.89135

**Published:** 2024-01-01

**Authors:** Lin Luo, Ping Li, QingZhi Xie, YunChou Wu, FuQiang Qin, DunMing Liao, Ke Zeng, KangNing Wang

**Affiliations:** 1 Department of Urology Surgery, The First Affiliated Hospital of Shaoyang University, Shaoyang City, Hunan Province, 422000, China.; 2 Department of Urology Surgery, Xiangya Hospital Central South University, Changsha City, Hunan Province, 410083, China.

**Keywords:** m6A, circFAM126A, miR-505-3p, Prostate cancer

## Abstract

Prostate cancer (PCa) is the most commonly diagnosed malignancy in men. In tumor biology, n6-methyladenosine (m6A) can mediate the production of circular RNAs (circRNAs). This study focused on the mechanism of m6A-modified circRNA family with sequence similarity 126, member A (FAM126A) in PCa. Cell counting kit-8 assay, colony formation assay, 5-ethynyl-2'-deoxyuridine assay, transwell assay, and xenograft mouse models were applied to study the role of circFAM126A in PCa cell growth and tumor metastasis, and cellular triglyceride and cholesterol levels were measured to assess cholesterol synthesis. RNA immunoprecipitation, RNA pull-down, luciferase reporter gene assay, and western blot were adopted to explore the underlying molecular mechanism. Data showed that circFAM126A was upregulated in PCa and promoted PCa progression *in vitro*. m6A modification of circFAM126A enhanced transcriptional stability. CircFAM126A targeted microRNA (miR)-505-3p to mediate calnexin (CANX). Up-regulating miR-505-3p or inhibiting CANX suppressed cholesterol synthesis and malignant progression in PCa cells. Overexpressing CANX suppressed the inhibitory effect of circFAM126A silencing or miR-505-3p upregulation on PCa cells. Our current findings provide a new therapeutic strategy for the treatment of PCa.

## Introduction

Prostate cancer (PCa) is the second leading cause of human malignancies worldwide 1 with increasing mortality and morbidity over the past 20 years 2. PCa remains a major medical challenge given that tumor resistance to radiotherapy, chemotherapy, or androgen deprivation therapy stimulates tumor metastasis and recurrence 3. PCa is a complex disease with the involvement of deregulated oncogenes and tumor suppressor genes 1. Therefore, elucidating the genetic mechanism of PCa is crucial to improve the efficiency of clinical treatment.

N(6)-methyladenosine (m6A) is the most abundant reversible methylation modification in eukaryotic RNA 4, 5. m6A modification is thought to control the production and functions of noncoding RNAs including circular RNA (circRNA) and microRNA (miRNA) 6, 7. CircRNAs are eukaryotic co-valently closed endogenous biomolecules with tissue-specific and cell-specific expression patterns 8, 9. Studies have shown that circRNAs can promote or inhibit tumorigenesis in various cancers, including hepatocellular carcinoma 10, breast cancer 11, glioblastoma 12, and bladder cancer 13. So far, some circRNAs have been reported to be associated with PCa progression, such as circ_0086722 14 and circRNA SR-related and CTD-associated factor 8 15. However, the function and underlying mechanism of circRNA family with sequence similarity 126, member A (circFAM126A) in PCa have not been thoroughly investigated.

By interacting with DNA, miRNA, lncRNA or protein, circRNA acts as a regulatory factor of various gene expression at different levels and plays a regulatory role in various cell physiological and pathological processes 16. miRNAs have been widely studied as key regulators in PCa 17-19, and multiple miRNAs have been identified as biomarkers for cancer diagnosis, treatment, and prognosis 20, 21. However, the mechanism of miR-505-3p in PCa remains unclear.

This study identified a novel circRNA, circFAM126A in the circMine dataset and explored the effect of circFAM126A on the biological characteristics of PCa cells. A hypothesis was put forward as follows: m6A-modified circFAM126A mediates miR-505-3p-targeted calnexin (CANX) to affect cholesterol synthesis and malignant progression in PCa.

## Materials and Methods

### Bioinformatics analysis

The GEO dataset (GSE113153) was analyzed via the Bioinformatics website circmine (http://www.biomedical-web.com/circmine/home) to assess differential circRNA in PCa. The gene screening criteria were Log2FC > 1, and the adjusted P value was < 0.05. Starbase website (https://rnasysu.com/encori/) was used to predict targeted binding sites between circFAM126A and CANX with miR-505-3p.

### Clinical samples

Clinical PCa tissue and normal tissue specimens were obtained from 46 newly diagnosed PCa patients who underwent prostatectomy in the First Affiliated Hospital of Shaoyang University from 2013 to 2018. These tumor specimens were microscopically evaluated by two experienced pathologists and classified into I + II stage and III + IV stage according to tumor node metastasis (TNM) stages (URL: www.cancerstaging.org/). No patient received preoperative anticancer therapy or chemoradiotherapy. The specimens were stored in liquid nitrogen. Survival data were collected for 5 consecutive years.

The inclusion and exclusion criteria for patients were as follows. Inclusion criteria: (1) Male patients aged between 50 and 80 years; (2) Histopathologically confirmed PCa patients; (3) Well organ function, such as kidney function and liver function. Exclusion criteria: (1) other major medical conditions such as serious heart disease, uncontrolled high blood pressure, and diabetes complications; (2) History of other malignancies within the last five years; (3) Severe mental illness or cognitive impairment, inability to understand study procedures or follow study guidelines; (3) Drug administration that may interfere with the results of the study, or allergy to drugs used in the study.

### Cell lines and cell culture

Human normal embryonic kidney cell line HEK293T, human normal prostate epithelial cell line RWPE1, and PCa cell lines (PC-3, DU145, LNCAP, VCAP, and 22RV1) were purchased from the National Collection of Authenticated Cell Cultures (Shanghai, China). HEK293T and VCAP cells were cultured in Dulbecco's modified Eagle medium (Gibco), RWPE-1 cells in keratinocyte serum-free medium (Gibco), and PC-3, DU145, LNCAP, and 22RV1 cells in Roswell Park Memorial Institute (RPMI)-1640 medium (Gibco). Fetal bovine serum (10%, Gibco), 100 U/ml penicillin, and 100 μg/ml streptomycin (Invitrogen) were supplementary to the above medium. The cells were cultured at 37°C in a humidified incubator with 95% air and 5% CO_2_ 22.

### Transfection

miR-505-3p mimic/inhibitor and its blank control (miR-NC), short hairpin RNAs targeting circFAM126A (sh-CircFAM126A#1 and sh-CircFAM126A#2) and its control (sh-NC) were purchased from GenePharma. CANX sequences were PCR-amplified and subcloned into pcDNA3.1 (Thermo Fisher Scientific) to obtain a CANX overexpression plasmid, named pcDNA-CANX. PC-3 and DU145 cells were transfected using Lipofectamine 2000 (Thermo Fisher Scientific) 23.

### Cell counting kit (CCK)-8 assay

Cell proliferation was assessed with CCK-8 (Dojindo, Kumamoto, Japan) Cells were cultured for 24, 48, and 72 h in 96-well plates, and the medium was replaced with 10 μl CCK-8 solution in each well. After 2 h, optical density values were read at 450 nm on a Varioskan™ LUX microplate reader (Thermo Fisher Scientific).

### Colony formation test

Cells were seeded into 6-well plates (700 cells/well) and cultured in an incubator containing 5% CO_2_ at 37℃ for 14 d. The colonies were fixed with 4% paraformaldehyde (Beyotime) for 10 min and stained with 0.1% crystal violet solution (Beyotime) for 5 min. Colonies containing at least 50 cells were counted 24.

### 5-ethynyl-2'-deoxyuridine (EdU) assay

The proliferation of PCa cells was detected by EdU staining proliferation kit (Abcam, USA). Cells were incubated with EdU solution for 24 h and added with the fixative for 15 min. Then, cells were added with a permeability buffer for 15 min, marked with fluorescently-labeled EdU solution for 30 min, and observed under a fluorescence microscope 25. EdU-positive cells were counted using the ImageJ software (National Institutes of Health).

### Cell migration and invasion assays

Cells were suspended in a serum-free RPMI-1640 medium. The suspended cells were seeded at a density of 10^5^ cells/well to the filter (with or without Matrigel) in the upper chamber of 8.0-μm transwell plate (Corning). After 72-96 h, migrating and invading cells on the lower surface of the filter were fixed in methanol and stained with 0.05% crystal violet solution. The stained cells were counted in four randomly selected fields of view using Olympus CKX52 microscope (magnification, × 200).

### Flow cytometry

Apoptosis was detected by Annexin apoptosis assay kit (Sigma) according to the prescribed procedure. At 48-h post-transfection, cells were resuspended in a binding buffer, added with 5 μL Annexin V-fluorescein isothiocyanate and 5 μL propidium iodide in the dark for 5 min, and analyzed by tune NxT (Thermo Fisher Scientific) 26.

### Measurement of triglyceride (TG) and cholesterol levels

Cells were lysed using the kit (Nanjing Jiancheng), in which TG and cholesterol levels were quantified with quantification kits, respectively 27.

### RNA extraction, RNase R treatment, polymerase chain reaction (PCR) detection

Total RNA extracts were collected from cells and tissues using RNeasy Mini Kit (QIAGEN, Germany). complementary DNA (cDNA) was synthesized using random primer and PrimeScript RT Master Mix reverse transcription kits (Takara, Dalian, China) or miRNA reverse transcription PCR kits (Ribo-Bio). To isolate genomic DNA, QIAamp DNA Mini kits (QIAGEN) was used. PCR analysis was done with SYBR Premix Ex Taq^TM^ kits (Takara). circRNA and miRNA were normalized to glyceraldehyde-3-phosphate dehydrogenase (GAPDH) or U6 levels, respectively. Data were analyzed in the StepOnePlus real-time PCR system (Applied Biosystems). Bulge-loop miRNA qPCR primers (RiboBio) were seen in Table 1 28.

### Western blot

Proteins from cells and tissues were collected using radioimmunoprecipitation assay lysis buffer (Thermo Fisher Scientific) and analyzed for protein concentration using the bicinchoninic acid protein assay kit (Pierce, USA). Then, proteins (30 μg) were electrophoresed on sodium dodecyl sulfate polyacrylamide gel, electroblotted onto polyvinylidene fluoride membranes, and blocked with 5% nonfat milk for 2 h in tris-buffered saline Tween-20. Primary antibodies against CANX (1:1000, ab22595, Abcam), insulin-like growth factor 2 mRNA-binding protein 1 (IGF2BP1, 1:1000, ab82968, Abcam), Vascular endothelial growth factor (VEGF, 1:1000, sc-7269, Santa Cruz Biotechnology), programmed death-ligand 1 (PD-L1, 1:1000, 13684, Cell Signaling Technology), and GAPDH (1:1000; ab37168, Abcam) were incubated with the membrane at 4°C overnight, and horseradish peroxidase-linked secondary antibody (1:500; SC-2054; Santa Cruz Biotechnology) was added for 2 h at room temperature. Protein bands were visualized using an enhanced chemiluminescence detection kit (Millipore) 29.

### RNase R test

Total RNA from PC-3 and DU145 cells was treated with RNase R (Sigma) for 15 min at 37℃ and then purified with phenol-chloroform (Sigma) to measure circular or linear FAM126A by reverse transcription quantitative PCR (RT-qPCR).

### Actinomycin D test

Actinomycin D (5 µg/mL) was added to the culture medium of cells and incubated for 0, 4, 8, 16, and 24 h. The stability of mRNA was analyzed by PCR 30.

### Methylated RNA immunoprecipitation (MeRIP)-qPCR

m6A modification was measured by Magna MeRIP™ m6A Kit (Millipore). Briefly, 150 μg RNA extracted from pretreated cells were prepared to fragments (≤ 100 nt) and immunoprecipitated with magnetic beads coated with 10 μg anti-m6A (Millipore) or anti-mouse immunoglobulin G (Millipore). m6A enrichment was normalized 31.

### Fluorescence in situ hybridization (FISH)

The subcellular location ofcircFAM126A1 was determined by FISH. Hybridization was done using RNA FISH kit and three 5'-cy3-labeled probes targeting the splicing site of circFAM126A1 (GenePharma), during which the probe mixture was concentrated at 8 μmol/L. Photography was done under a fluorescence inverted microscope (Olympus) 32.

### RNA-binding protein immunoprecipitation (RIP)

Cells after 48-h transfection were lysed in the RIP lysis buffer on ice for 30 min and centrifuged. The supernatant was incubated with antibodies and 30 μl Protein-A/G agarose beads (Roche) overnight. The immune complexes were centrifuged, washed with cleaning buffer 6 times, and analyzed by RT-qPCR.

### RNA pull-down assay

Cell lysates were incubated with streptavidin (Invitrogen)-coated magnetic beads to pull down biotin-conjugated RNA complexes. The enrichment of circFAM126A1 was assessed by RT-qPCR analysis. Bound proteins were eluted from the packaged beads and analyzed by sodium dodecyl sulfate-polyacrylamide gel electrophoresis.

### Xenograft model

PC-3 cells after digestion and centrifugation were resuspended in phosphate buffered saline (PBS) to 1 × 10^6^/mL and subcutaneously injected into BALB/c nude mice (n = 6) at a dose of 0.1 mL. The same dose of PBS served as a blank control. Tumor nodules that appeared at the injection site were measured with a vernier caliper every week. The longest diameter (a) and longitudinal diameter (b) were recorded to calculate tumor volume (a × b^2^/2). After 4 weeks, tumors were excised from euthanized mice (3% pentobarbital sodium, intraperitoneal injection, 160 mg/kg) and weighed 33.

### Immunohistochemistry

Xenografts were cryopreserved and sectioned to 5 μm by Tek O.C.T (Fisher Scientific). Cleaved caspase-3 (9661, Cell Signaling Technology) and Ki-67 (ab15580, Abcam) were added to the sections overnight at 4°C. Subsequently, the sections were added with horseradish peroxidase-labeled secondary antibody and incubated at 37℃ for 30 min. Streptavidin-biotin complex was added at 37℃ for 20 min, and the sections were developed with diaminobenzidine. Sections were stained with hematoxylin, dehydrated and permeated with xylene, and sealed with neutral balsam. Staining intensity and staining positive cells were analyzed using the ImageJ software (National Institutes of Health) 34.

### Oil red O staining

Oil Red O solution (6 mL, Sigma-Aldrich) and distilled water (4 mL) were mixed. After 10 min, the mixture was dropped on the xenografts for 5-10 min, and excessive staining buffer was removed with 60% Oil Red O and isopropanol solution. The tumors were rinsed with distilled water and counterstained with hematoxylin (Servicebio, Wuhan, China) 35.

### Lung metastases

PC-3 cells (2 × 10^6^) that were transfected with LV-sh-circFAM126A or LV-sh-NC for 48 h were suspended in 0.1 ml PBS and injected into the caudal vein of mice. Then, mice were isoflurane-anesthetized and given an intraperitoneal injection with D-luciferin (15 mg/ml PBS) at 150 mg/kg. After 7 weeks, D-luciferin injection was repeated once again. After 10 min, bioluminescence signals in mice were captured with a high-sensitivity camera in a IVIS200 chamber (Xenogen) and quantified by live Image software (Xenogen) 36.

### Statistical analysis

SPSS 21.0 and Prism 6.0 were needed to analyze data. Measurement data (mean ± standard deviation) were compared by t test and one-way ANOVA. For correlation analysis, Pearson method was utilized. All tests were two-sided and *p* < 0.05 was considered statistically significant.

## Results

### circFAM126A expression pattern in PCa

**Figure 1:**
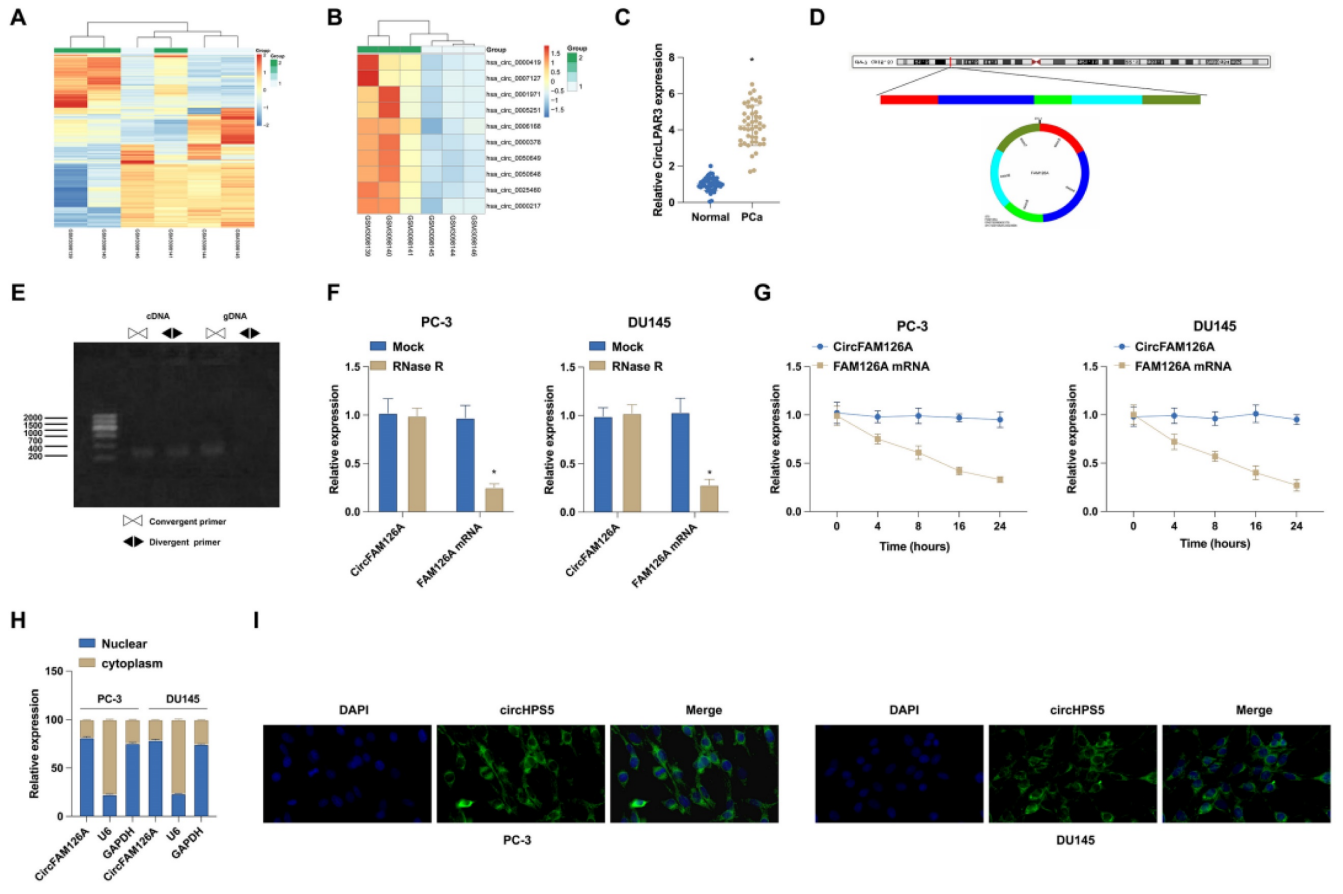
** circFAM126A expression in PCa.** A and B: Clustering and scatter plots from circRNA microarray data exhibit the differential expression of circRNAs in PCa groups compared to normal groups, with red indicating high expression levels and green signaling low expression levels. C: The expression of hsa_circ_0001971 in 46 pairs of matched PCa and adjacent non-tumor tissues was detected using RT-qPCR. D: Genomic locus and circular structure of hsa_circ_0001971. E: The presence of hsa_circ_0001971 was validated using divergent and convergent primers for cDNA and gDNA. F and G: RT-qPCR experiments were conducted to assess the effect of RNase R and Actinomycin D on the RNA stability of CircFAM126A and its linear counterpart, FAM126A, in cells. H: The subcellular localization of CircFAM126A was determined through nuclear and cytoplasmic separation experiments. I: FISH experiments revealed that CircFAM126A is predominantly localized in the cytoplasm. Data are expressed as mean ± SD (n = 3), with * *P* < 0.05 indicating statistical significance.

The circRNA transcripts of 3 PCa tissue samples with Gleason score < 6 and 3 PCa tissue samples with Gleason score > 8 (circmine ID: HSACM000016) were obtained from circMine (http://www.biomedical-web.com) (Fig. 1A). In total, 862 circRNAs were upregulated and 497 circRNAs were downregulated (Fig. 1B). Since hsa_circ_0001971 expression exhibited 3.7754 log2 fold change, it was selected as the circRNA of interest. To further validate the RNA-seq data, hsa_circ_0001971 in 46 paired PCa tissues and normal tissues was measured, and it was found that hsa_circ_0001971 expression was elevated in PCa tissues (Fig. 1C). According to the circBase information database (http://www.circbase.org/), the mature sequence of hsa_circ_0001971 after splicing is located at chr12:70193988-70195501 with a length of 1513 bp, which contains exons 3-7 from FAM126A mRNA (Fig. 1D). The ring structure and subcellular localization of circFAM126A were then evaluated by cell experiments. PCR analysis validated that circFAM126A could be amplified by different primers amplified from random hexamer reverse-transcribed cDNA, whereas not by gDNA primers (Fig. 1E). Resistance of circFAM126A to RNase R digestion confirmed that circFAM126A has a closed-loop structure (Fig. 1F). Actinomycin D treatment showed a more stable half-life of circFAM126A transcripts over 24 h compared to FAM126A (Fig. 1G). According to nuclear and cytoplasmic separation experiment results, circFAM126A was distributed in both the nucleus and cytoplasm, and the distribution in the cytoplasm was higher than that in the nucleus (Fig. 1H). This finding was further verified by FISH (Fig. 1I). These data confirm that circFAM126A is abnormally high expressed in PCa as a circRNA, which may be involved in regulating PCa occurrence and development.

### circFAM126A is associated with poor prognosis in PCa

**Figure 2:**
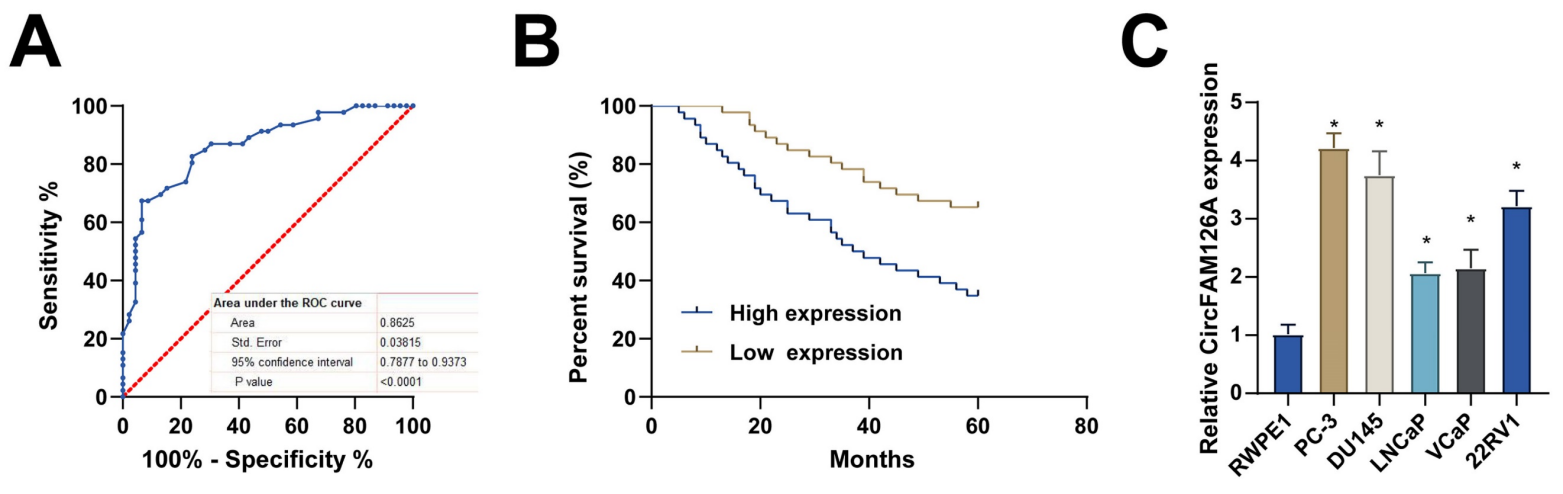
** circFAM126A is associated with poor prognosis in PCa.** A: ROC curve analysis was employed to evaluate the proficiency of circFAM126A in distinguishing between PCa tissues and normal tissues. B: Kaplan-Meier survival curves were used to assess the relationship between CircFAM126A expression and the survival prognosis of PCa patients. C: The expression levels of CircFAM126A in the normal human prostate epithelial cell line RWPE1 and various PCa cell lines (PC-3, DU145, LNCAP, VCAP, and 22RV1) were quantified using RT-qPCR. Data are presented as mean ± SD (n=3), with * *P* < 0.05 indicating statistical significance.

Taking circFAM126A medium expression as a cutoff value, PCa patients were allocated to the circFAM126A high-expression group and circFAM126A low-expression group. Analysis of clinicopathological features found that circFAM126A high expression was positively correlated with tumor size, TNM stage, and microvascular invasion (Table 2). Receiver operating characteristic curve analysis calculated that the area under the curve of circFAM126A to differentiate PCa tissue from normal tissue was 0.8625 (Fig. 2A). Kaplan Meier survival curves showed that patients with higher circFAM126A expression had shorter overall survival (Fig. 2B). RT-qPCR measured that circFAM126A was significantly upregulated in PCa cells compared to the normal prostate epithelial cell line RWPE1 (Fig. 2C). PC-3 and DU145 cells with the most significant expression were selected for follow-up experiments.

### circFAM126A promotes PCa *in vitro*

**Figure 3:**
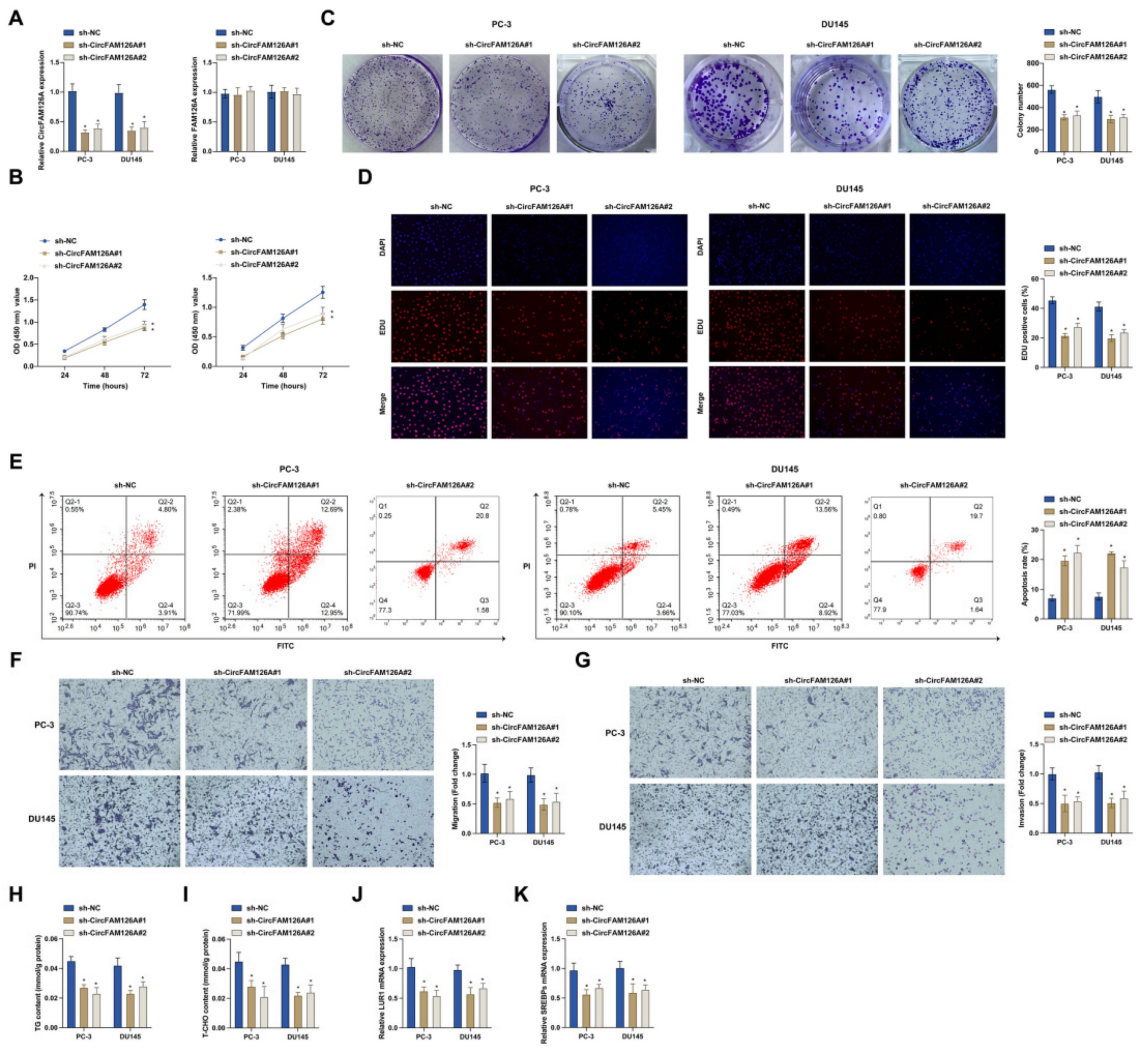
** circFAM126A promotes PCa *in vitro*.** sh-circFAM126A was transfected into PC-3 and DU145 cells to study its effects. A: The knockdown efficiency of sh-circFAM126A#1 and sh-circFAM126A#2 on circFAM126A was evaluated using RT-qPCR. B: The cell proliferation rate was measured using the CCK-8 assay. C: Colony formation assays were performed to assess cell proliferation capability. D: EdU assay was utilized to determine the Edu-positive rate in cells. E: Flow cytometry was employed to analyze cell apoptosis. F and G: Transwell assays were conducted to investigate the cell migration and invasion abilities. H and I: TG and cholesterol levels in cells were measured using commercial assay kits. J and K: The mRNA levels of LUR1 and SREBPs in cells were detected via RT-qPCR. Data are presented as mean ± SD (n=3), with * *P* < 0.05 indicating statistical significance.

shRNAs were produced to silence circFAM126A without affecting FAM126A mRNA levels in PC-3 and DU145 cells (Fig. 3A). Data collected from CCK-8, colony formation assay, and EdU assay indicated that sh-circFAM126A suppressed PC-3 and DU145 cell proliferation (Fig. 3B-D). Then, flow cytometry detected that PC-3 and DU145 cell apoptosis was enhanced after knocking down circFAM126A (Fig. 3E). Next, Transwell assays found that silencing circFAM126A reduced the invasion and migration ability of PC-3 and DU145 cells (Fig. 3F, G). The importance of abnormal cholesterol metabolism in tumor cell physiology has been emphasized. TG and cholesterol levels were reduced in PC-3 and DU145 cells after inhibiting circFAM126A (Fig. 3H, I). LUR1 (also called C12orf49) is a novel regulator of lipid production, which regulates the mRNA processing of sterol regulatory element-binding proteins (SREBPs) to up-regulate SREBPs and accelerate cholesterol synthesis. LUR1 and SREBPs mRNA levels were decreased after inhibition of circFAM126A (Fig. 3J, K). These results suggest that inhibition of circFAM126A inhibits cholesterol synthesis and malignant progression of PCa cells.

### m6A modification of circFAM126A improves transcriptome stability

**Figure 4:**
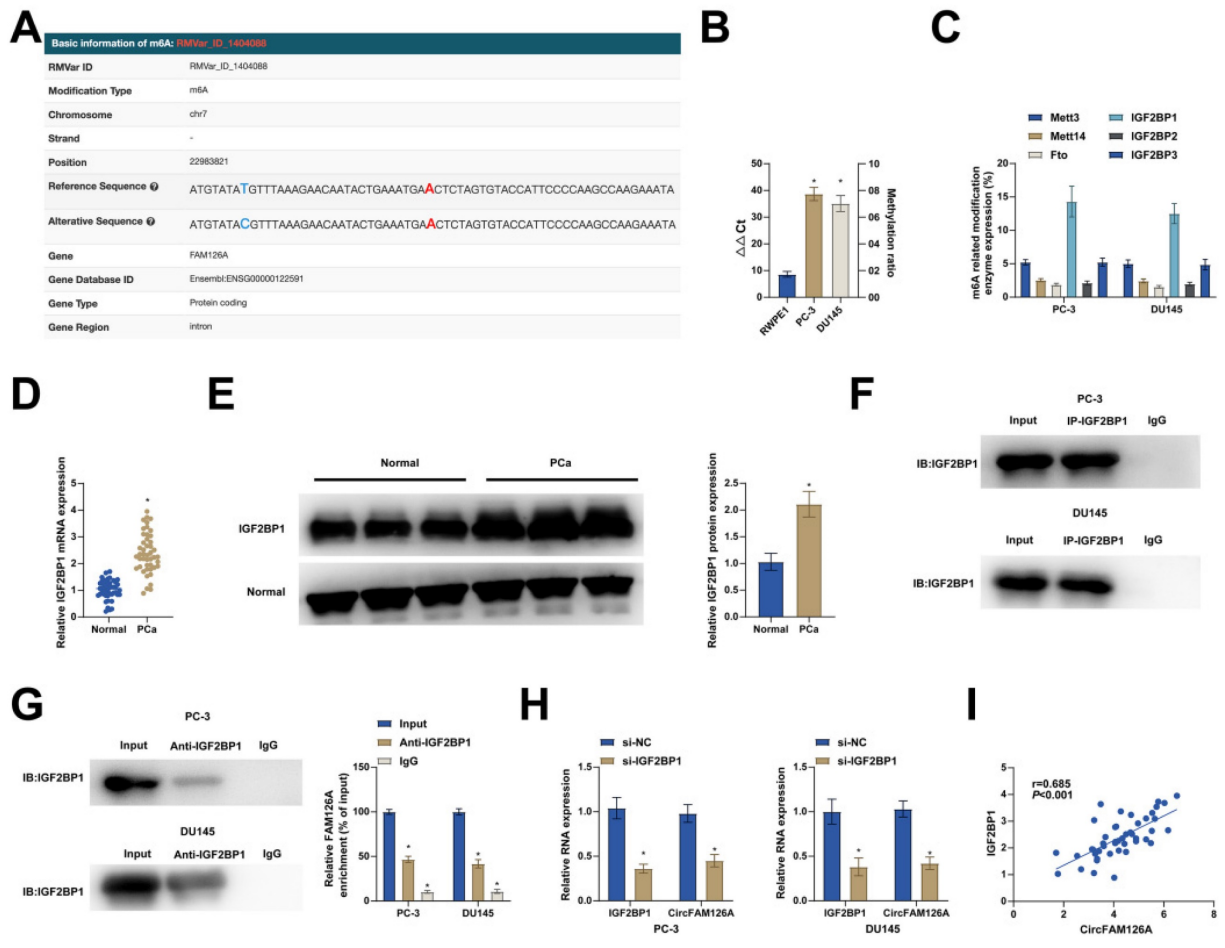
** m6A modification of circFAM126A improves transcriptome stability.** A: The bioinformatics tool m6A Avar revealed the presence of m6A modification sites on CircFAM126A. B: MazF-PCR was employed to assess the abundance of m6A-modified CircFAM126A in cells. C: The expression levels of methylation-related proteins were quantified using RT-qPCR. D: RT-qPCR was utilized to measure the mRNA levels of IGF2BP1 in 46 paired PCa samples. E: Western Blot analysis was conducted to evaluate the protein expression of IGF2BP1 in 46 paired PCa samples. F: RNA pull-down assays were performed to investigate the targeted binding relationship between IGF2BP1 and CircFAM126A. G: RIP assays were employed to confirm the targeted interaction between IGF2BP1 and CircFAM126A. H: The effects of IGF2BP1 knockdown on IGF2BP1 and CircFAM126A levels in cells were assessed using RT-qPCR. I: Pearson correlation analysis demonstrated a positive correlation between CircFAM126A and IGF2BP1 mRNA levels in the tumor tissues of 46 PCa patients. Data are presented as mean ± SD (n = 3), with * *P* < 0.05.

In order to explore m6A modification of circFAM126A, m6A site in circFAM126A was predicted using the online bioinformatics tool m6A Avar (http://m6avar.renlab.org/) (Fig. 4A). MazF was used to provide nucleotide resolution quantification of m6A methylation sites. MazF toxin is an aca sequence-specific endorbonuclease that is sensitive to the m6A site and represents the first m6A sensitive RNA lyase. m6A levels were higher in PC-3 and DU145 cells than in RWPE1 cells (Fig. 4B). Then, Mett3, Mett14, Fat mass and obesity associated (Fto) gene, IGF2BP1, nsulin-like growth factor 2 mRNA-binding protein 2 (IGF2BP2), and nsulin-like growth factor 2 mRNA-binding protein 3 (IGF2BP3) in PC-3 and DU145 cells were examined, and it was found that IGF2BP1 was mostly expressed, suggesting that IGF2BP1 protein is the m6a-binding protein of circFAM126A (Fig. 4C). In clinical samples, Western blot and RT-qPCR measured that IGF2BP1 was highly expressed in PCa tissues (Fig. 4D, E). To further investigate the interaction of circFAM126A with IGF2BP1, RNA pull-down was conducted. It was found that circFAM126A probe was enriched for circFAM126A and IGF2BP1 (Fig. 4F), and significant enrichment of circFAM126A was observed in IGF2BP1 immunoprecipitants by anti-IGF2BP1 (Fig. 4G). Furthermore, knockdown of IGF2BP1 resulted in decreased expression of circFAM126A (Fig. 4H). Correlation analysis also showed that circFAM126A was positively correlated with IGF2BP1 (Fig. 4I). Taken together, the m6A reading protein IGF2BP1 can bind to circFAM126A *in vitro*, and that m6A modification enhances the transcriptome stability of circFAM126A, which may be part of the reason for the significant up-regulation of circFAM126A in PCa.

### circFAM126A as a sponge for miR-505-3p

**Figure 5:**
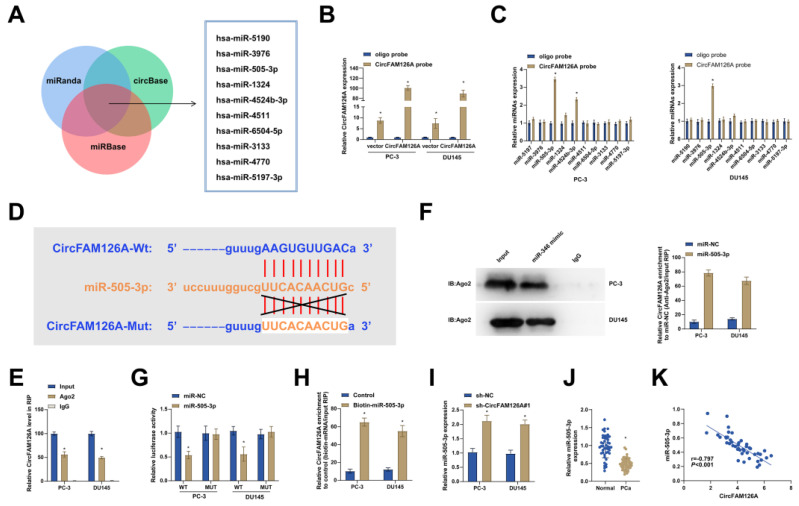
** circFAM126A as a sponge for miR-505-3p.** A: The potential miRNAs of CircFAM126A were predicted using bioinformatics websites such as miRanda, miRBase, and circBase. B: Lysates of PCa cells overexpressing CircFAM126A were subjected to a biotinylated pull-down assay, and the expression levels of CircFAM126A were detected by RT-qPCR. C: Candidate miRNAs of circFAM126A were analyzed using the biotinylated pull-down assay. D: The bioinformatics website Starbase predicted potential binding sites between CircFAM126A and miR-505-3p. E and F: Ago2-RIP assays confirmed the targeting relationship between miR-505-3p and circFAM126A. G: Dual-luciferase reporter assays were performed to investigate the targeting relationship between miR-505-3p and circFAM126A. H: The targeting relationship between miR-505-3p and circFAM126A was analyzed through a biotinylated pull-down assay. I: The impact of CircFAM126A knockdown on the expression of miR-505-3p was determined by RT-qPCR. J: The expression of miR-505-3p in cancer tissues and adjacent normal tissues was measured by RT-qPCR. K: Pearson correlation analysis indicated a negative correlation between CircFAM126A and miR-505-3p levels in the tumor tissues of 46 PCa patients. Data are represented as mean ± SD (n = 3), with * *P* < 0.05 denoting statistical significance.

Considering the cytoplasmic distribution of circFAM126A, it was speculated that circFAM126A may function by targeting miRNAs. Potential targets of circFAM126A were predicted using miRNA target prediction tools such as miRDB, miRanda and circBase. Among 246 candidate miRNAs overlapping in the 3 databases, the top 10 miRNAs were selected for further analysis (Fig. 5A). To validate our predictions, biotinylated circFAM126A probes were designed and the pull-down efficiency was confirmed in PCa cells overexpressing circFAM126A (Fig. 5B). circFAM126A probe pulled down miR-505-3p in PC-3 and DU145 cells (Fig. 5C). The binding site between circFAM126A and miR-505-3p waspredicted by Starbase (Fig. 5D). To further verify the direct binding of miR-505-3p to circFAM126A, RIP experiments were conducted. It was found that circFAM126A was preferentially enriched in ago2-RIP (Fig. 5E). In addition, Ago2-RIP experiment also reported that circFAM126A was enriched in the miR-505-3p mimic group (Fig. 5F). Subsequently, dual-luciferase reporter experiments also manifested that overexpression of miR-505-3p reduced the luciferase activity of the wild-type circFAM126A reporter gene, but not that of the mutant circFAM126A reporter (Fig. 5G). In addition, biotin-labeled miRNA pull-down experiments proved that circFAM126A was elevated in PCa cells transfected with biotin-labeled miR-505-3p (Fig. 5H). At the same time, circFAM126A silencing increased miR-505-3p levels (Fig. 5I). In addition, downregulated miR-505-3p was also detected in tumor tissues of 46 PCa patients (Fig. 5J) and was negatively correlated with circFAM126A (Fig. 5K). These results suggest that circFAM126A directly sponges miR-505-3p.

### miR-505-3p inhibits the malignant progression of PCa cells

**Figure 6:**
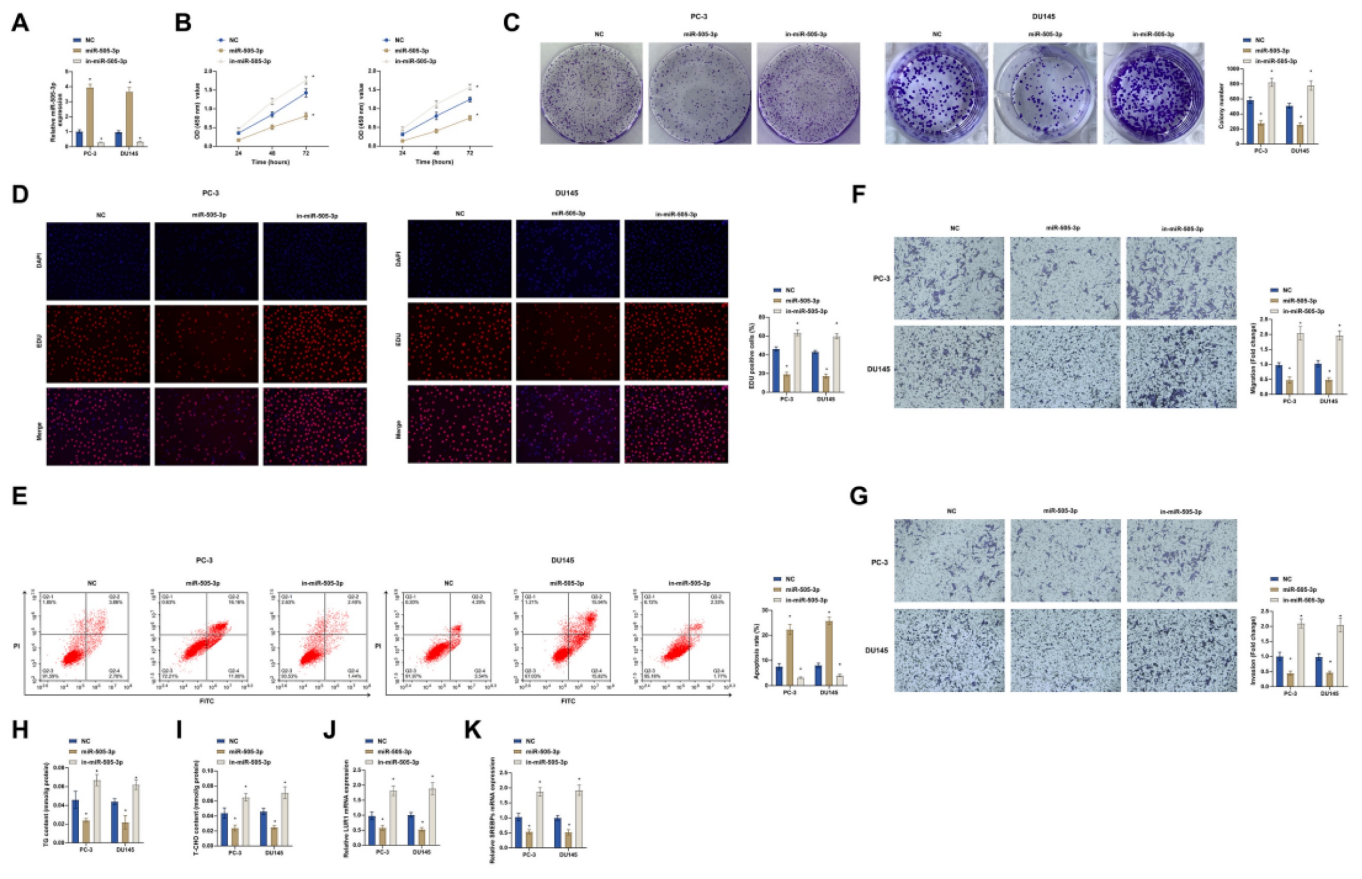
** miR-505-3p inhibits the malignant progression of PCa cells.** miR-505-3p mimic/inhibitor was transfected into PC-3 and DU145 cells. A: The transfection efficiency of the miR-505-3p mimic/inhibitor was evaluated using RT-qPCR. B: Cell proliferation rates were measured by the CCK-8 assay. C: Colony formation assays were conducted to assess cell proliferative capacity. D: EdU assay was utilized to determine the proportion of Edu-positive cells, indicating DNA synthesis. E: Flow cytometry was performed to analyze cell apoptosis. F and G: Transwell assay was employed to examine cell migration and invasion abilities. H and I: Cellular levels of triglycerides (TG) and cholesterol were quantified using commercial assay kits. J and K: mRNA levels of LUR1 and SREBPs in the cells were detected through RT-qPCR. Data are represented as mean ± SD (n = 3), with * *P* < 0.05 denoting statistical significance.

Next, the role of miR-505-3p in PCa was further studied. PC-3 and DU145 cells were transfected with miR-505-3p mimic and inhibitor. miR-505-3p mimic elevated miR-505-3p expression, and miR-505-3p inhibitor reduced miR-505-3p expression (Fig. 6A). Functional experiments found that increasing miR-505-3p obstructed PC-3 and DU145 cell proliferation (Fig. 6B-D) and promoted apoptosis (Fig. 6E). In addition, up-regulation of miR-505-3p significantly reduced the invasion and migration capacity of cells (Fig. 6F, G). TG and cholesterol levels were also reduced (Fig. 6H, I). RT-qPCR analysis found that LUR1 and SREBPs mRNA expression were suppressed in PC-3 and DU145 cells overexpressing miR-505-3p (Fig. 6J, K). Low expression of miR-505-3p had the opposite results (Fig. 6B-K). These results indicated that overexpression of miR-505-3p inhibited the malignant progression of PCa cells.

### circFAM126A sponges miR-505-3p to upregulate CANX

**Figure 7:**
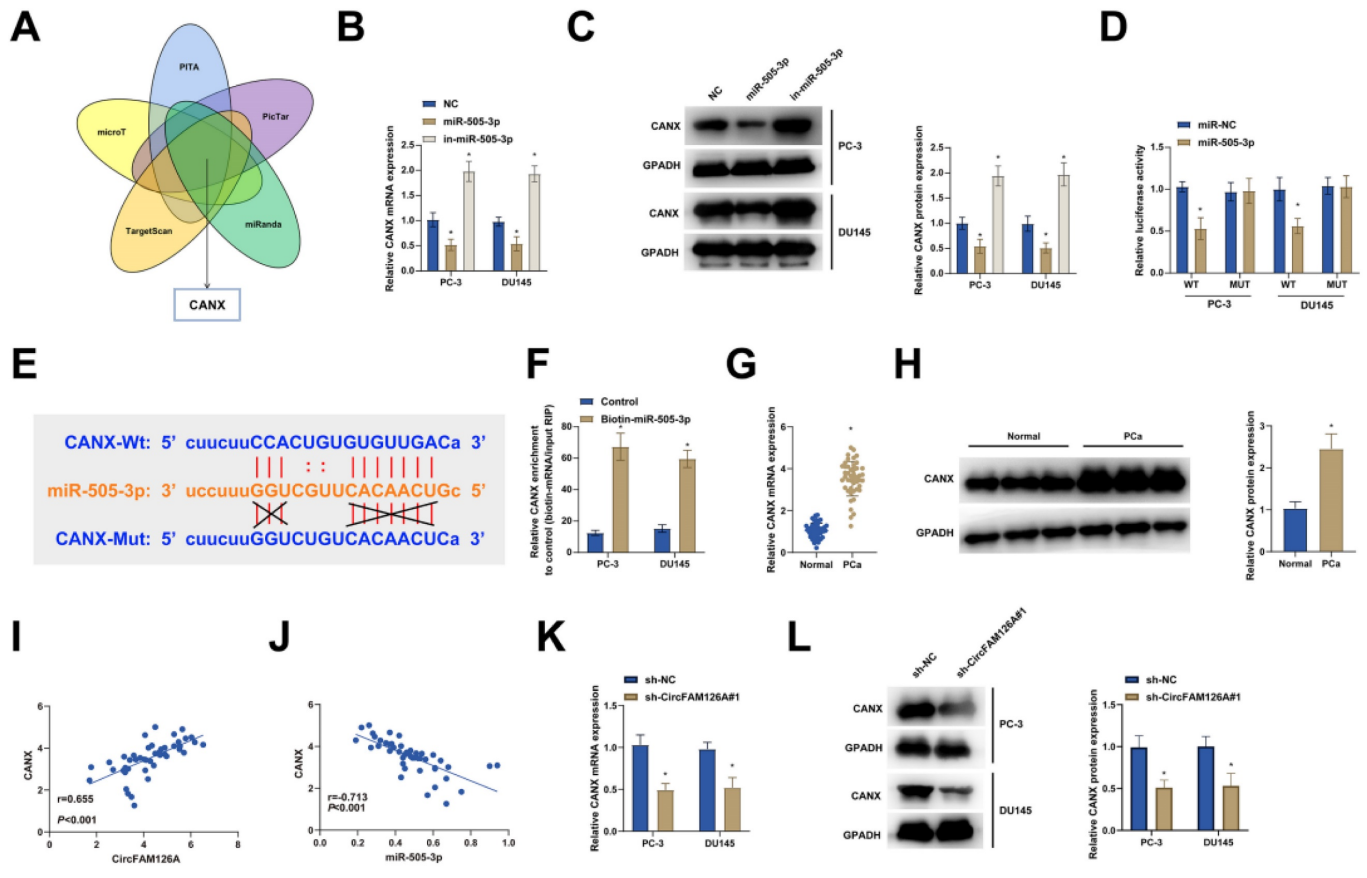
** circFAM126A sponges miR-505-3p to upregulate CANX.** A: Bioinformatics websites PITA, microT, TargetScan, PicTar, and the miRanda database were utilized to predict potential downstream mRNA targets of miR-505-3p. B and C: The impact of knocking down or overexpressing miR-505-3p on the mRNA and protein expression of CANX was examined using RT-qPCR and Western blot. D: Dual-luciferase reporter assay was conducted to investigate the target relationship between miR-505-3p and CANX. E: The bioinformatics website starbase was employed to predict potential binding sites between CANX and miR-505-3p. F: Biotinylated pull-down assay was used to analyze the targeting relationship between miR-505-3p and CANX. G and H: RT-qPCR and Western blot assays were carried out to measure the mRNA and protein expression of CANX in PCa tissues and adjacent normal tissues. I and J: Pearson correlation analysis was performed to assess the relationship among CircFAM126A, miR-505-3p, and CANX in cancer tissues. K and L: The effect of knocking down CircFAM126A on the mRNA and protein levels of CANX was detected using RT-qPCR and Western blot. Data are presented as mean ± SD (n = 3), with * *P* < 0.05 indicating statistical significance.

Target genes of miR-505-3p were predicted using PITA, MicroCosm, TargetScan, PicTar and miRanda, the intersection of these databases indicated that CANX was the most likely miR-505-3p target gene (Fig. 7A). RT-qPCR and Western Blot showed that regulating miR-505-3p could affect CANX expression (Fig. 7B, C). The assay of luciferase reporter gene showed that in PCa cells transfected with wild-type CANX 3'-UTR reporter, overexpression of miR-505-3p could inhibit the luciferase activity of wild-type CANX 3'-UTR reporter but had no influence on that of corresponding mutant reporter (Fig. 7D). Binding sites between CANX and miR-505-3p were predicted (Fig. 7E). In addition, biotin-labeled miRNA pull-down experiments confirmed that CANX was a target gene of miR-505-3p (Fig. 7F). RT-qPCR and Western Blot showed that CANX was highly expressed in PCa tissues (Fig. 7G, H). CANX expression was in a positive correlation with circFAM126A expression and in a negative correlation with miR-505-3p expression (Fig. 7I, J). Moreover, silencing circFAM126A reduced CANX levels (Fig. 7K, L). These findings suggest the presence of the circFAM126A-miR-505-3p-CANX regulatory axis.

### CANX overexpression re-activates cancer malignancy after inhibition of circFAM126A or overexpression of miR-505-3p

**Figure 8:**
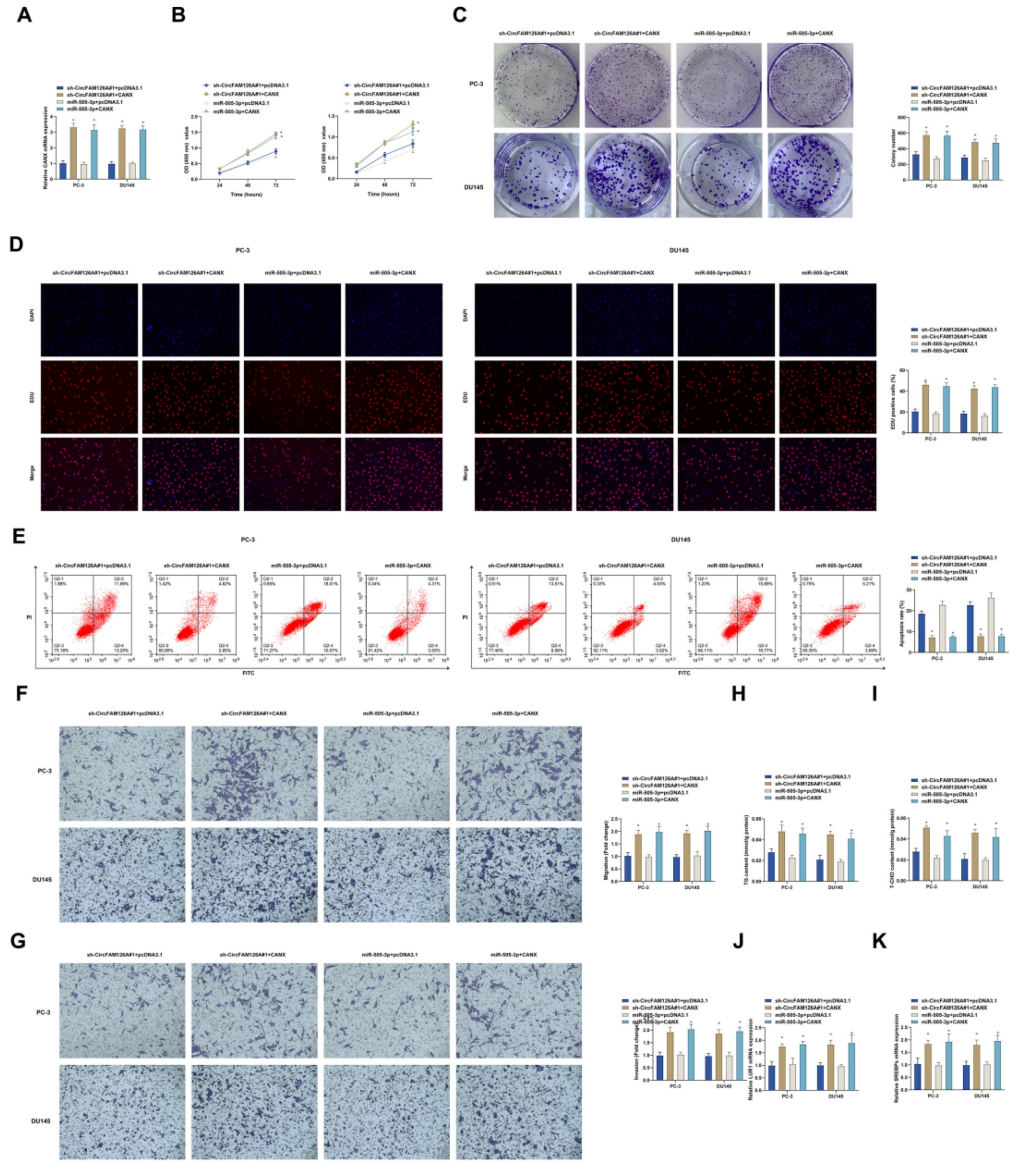
** CANX overexpression re-activates cancer malignancy after inhibition of circFAM126A or overexpression of miR-505-3p.** Sh-circFAM126A and miR-505-3p mimic were co-transfected into PC-3 and DU145 cells. A: The impact of co-transfection on miR-505-3p levels in cells was measured using RT-qPCR. B: Cell proliferation rate was assessed through the CCK-8 assay. C: Colony formation assay was conducted to evaluate the cell proliferation capacity. D: EdU incorporation was used to detect the rate of Edu-positive cells. E: Flow cytometry was performed to examine cell apoptosis. F and G: Transwell assays were utilized to assess the migration and invasion capabilities of the cells. H and I: Commercial kits were employed to measure the levels of TG and cholesterol in the cells. J and K: mRNA levels of LUR1 and SREBPs in the cells were detected by RT-qPCR. Data are presented as mean ± SD (n = 3), with * *P* < 0.05 indicating statistical significance.

The relationship between circFAM126A/miR-505-3p/CANX was then evaluated by functional rescue experiments. Transfection of pcDNA 3.1-CANX in cells that knocked down circFMA126A or overexpressed miR-505-3p promoted CANX expression (Fig. 8A). CCK-8, colony formation assay, and EdU assay showed that the inhibitory effect of circFMA126A knockdown or miR-505-3p overexpression on cell proliferation was suppressed by overexpression of CANX (Fig. 8B-D). Flow cytometry showed that the promoting effect of circFMA126A knockdown or miR-505-3p overexpression on cancer apoptosis rate was reversed by overexpression of CANX (Fig. 8E). Transwell experiments showed that transfection of pcDNA 3.1-CANX in cells that knocked down circFMA126A or overexpressed miR-505-3p restored the invasion and migration ability of cells (Fig. 8F, G). Furthermore, the inhibitory effects of circFMA126A knockdown or overexpression of miR-505-3p on TG and cholesterol were reversed by overexpression of CANX (Fig. 8H, I). RT-qPCR experiments showed that the inhibitory effect of circFMA126A knockout or overexpression of miR-505-3p on mRNA expression of LUR1 and SREBPs was blocked by overexpression of CANX (Fig. 8J, K). These results indicate that circFAM126A and miR-505-3p affect the malignant behavior of PCa by targeting CANX.

### Silencing circFAM126A inhibits PCa xenograft tumor formation

**Figure 9:**
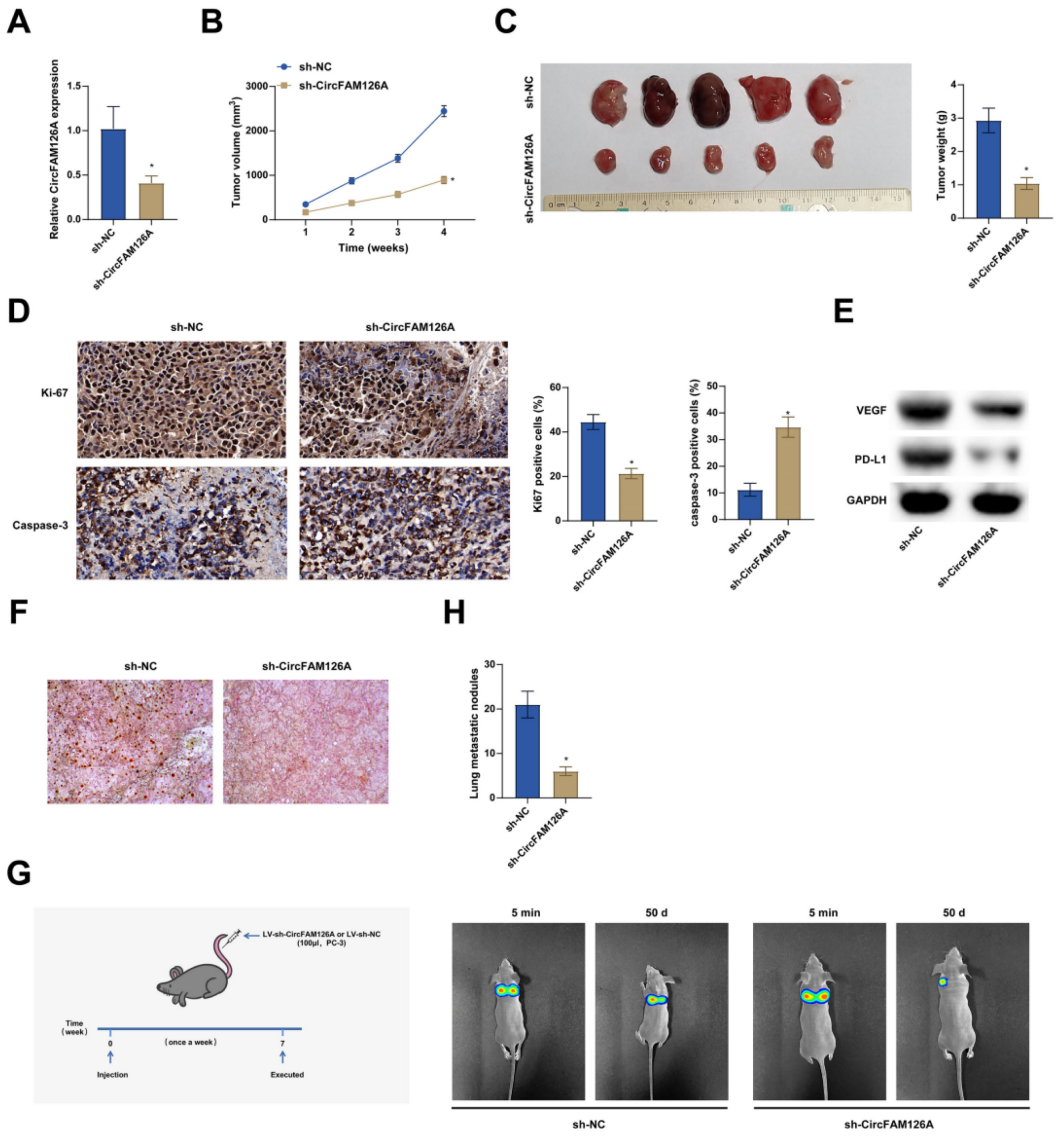
** Silencing circFAM126A inhibits PCa xenograft tumor formation.** A: The expression of CircFAM126A in tumors was investigated using RT-qPCR. B and C: Measurements were taken for both the volume and weight of the tumors to assess their growth. D: Immunohistochemical staining techniques were applied to detect the cellular proliferation marker Ki67 and the activated apoptosis indicator cleaved caspase-3 in tumor tissues. E: The protein levels of VEGF and PD-L1 in tumor samples were examined using Western blot analysis. F: Oil Red O staining was utilized to quantify the lipid content present in lung tissues. G: Bioluminescence imaging provided a visualization of the metastatic spread of cancer cells to the lungs. H: A quantitative assessment was made regarding the lymph nodes within lung tissues. Data are presented as mean ± SD (n = 6), with * *P* < 0.05 indicating statistical significance.

By subcutaneous injection of PCa cells transfected with pre-constructed LV-sh-circFAM126A and LV-sh-NC into nude mice (Fig. 9A), the tumor-suppressing effect of circFAM126A downregulation was further confirmed. circFAM126A knockdown significantly inhibited tumor growth, as evidenced by the relatively small tumor size and weight (Fig. 9B, C). The proliferation and apoptosis of xenografts were analyzed by immunohistochemical staining of proliferation marker Ki-67 and apoptosis marker caspase-3. Immunohistochemical staining indicated that the number of Ki-67-positive cells decreased and that of caspase-3-positive cells increased in circFAM126A-low-expressing tumor xenografts (Fig. 9D). In addition, knockdown of circFAM126A significantly inhibited VEGF and PD-L1 protein expression in tumors, suggesting that circFMA126A has a positive effect on tumor angiogenesis and immune escape (Fig. 9E). Oil red O staining showed that lipid content was reduced after inhibition of CircFAM126A (Fig. 9F). PCa cells transfected with LV-Sh-circFAM126A or control were injected into the tail vein of nude mice to measure the effect of circFAM126A on tumor metastasis *in vivo*. Bioluminescence imaging showed that inhibition of circFAM126A prevented PCa cells from metastasis to the lung (Fig. 9G). After 7 weeks, anatomical observations revealed a marked reduction in the number of metastatic lymph nodes in nude mice injected with cells transfected with LV-sh-circFAM126A (Fig. 9H). These results highlight the role of CircFAM126A in PCa and suggest that inhibition of CircFAM126A inhibits tumor formation *in vivo*.

## Discussion

Various circRNAs are differentially expressed in various diseases, especially cancer. Its stability, abundance, conservation and spatiotemporal specificity make it a hot spot of biomedical research in recent years. This report identified differentially expressed circRNAs in PC and focused on circFAM126A in PCa. Compared with linear FAM126A, circFAM126A was stably expressed in PCa cells and was mainly localized to the cytoplasm, suggesting its role in post-transcriptional gene regulation. The expression and functional differences of circFAM126A may be related to the tissue specificity, suggesting that circFAM126A has the potential to serve as a promising prognostic biomarker to guide the development of personalized therapy for PCa patients.

CircRNAs are highly dysregulated in many types of cancer and exhibit highly tissue- and disease-specific 37. This study examined that circFAM126A was elevated in PCa tissues and PCa cell lines. Loss-of-function experiments suggest that circFAM126A was associated with tumor progression and circFAM126A knockdown reduced PCa cell malignancy. Abnormal cholesterol metabolism in tumor cell physiology has been appreciated 38. LUR1 is a novel lipid production regulator that upregulates SREBPs and accelerates cholesterol synthesis 39. This report found that TG and cholesterol levels were reduced, as well as mRNA expression of LUR1 and SREBPs in PC-3 and DU145 cells after circFAM126A inhibition. These tumor growth observations were validated in a mouse xenograft model and *in vivo* metastasis experiments.

The biogenesis mechanism of circRNAs is a very complex process 40. m6A has been confirmed to be an abundant transcription-related modification and is involved in the regulation of circRNAs, such as m6a-mediated upregulation of circMDK to promote tumorigenesis 41. m6A modifies circHPS5 to promote liver cancer progression 42. This study predicted m6A sites in circFAM126A. m6A RNA methylation is the most common internal mRNA modification in mammals, mediated by m6A methyltransferases, demethylases, or m6A-binding proteins 43. The study evidenced that IGF2BP1 protein is the m6a-binding protein of circFAM126A. In addition, m6A reader protein IGF2BP1 can bind circFAM126A in vitro, and m6A modification enhances the transcriptome stability of circFAM126A, which may be part of the reason why circFAM126A is upregulated in PCa.

CircRNAs exert their functions through a variety of biological processes, such as miRNA sponges 9. The ceRNA mechanism suggests that circRNAs competitively bind miRNAs to relieve the inhibition of miRNA-targeted genes 44. This study predicted the potential target of circFAM126A, miR-505-3p and further confirmed that miR-505-3p inhibited the growth, TG, and cholesterol in PCa. CANX is a chaperone protein involved in the folding and assembly of major histocompatibility class-I (MHC-I) molecules on the endoplasmic reticulum 45, 46. Aberrant expression of CANX prevents successful assembly of MHC-I, processing of antigenic peptides and presentation on the surface of tumor cells, thus potentially leading to evasion of immune surveillance 47. This study found that CANX was upregulated in PCa and acted as a target gene of miR-505-3p, and overexpression of CANX decreased the suppressive effects of circFAM126A and overexpression of miR-505-3p on cancer malignancy.

In conclusion, circFAM126A has an important role in PCa tumorigenesis and metastasis. Mechanistically, circFAM126A acts as a sponge for miR-505-3p and regulates FAM126A expression. Our study suggests that circFAM126A may be a potential biomarker and therapeutic target for PCa, enriching the research on the pathogenesis of PCa and providing a theoretical basis for in-depth exploration of the functions of circRNAs in PCa.

## Figures and Tables

**Table 1:** Sequence of all genes.

Genes		Sequence (5'-3')
CircFAM126A	Human	F: GCTGCCTTAACTTACATGCCC
		R: ACTTTGTGGCTCCTGGATAACT
	Mice	F: GCTGCCTTAACTTACATGCCC
		R: ACTTTGTGGCTCCTGGATAACT
miR-505-3p	Human	F: CGCGTCAACACTTGCTGG
		R: AGTGCAGGGTCCGAGGTATT
CANX	Human	F: GCTAAGAGGCCAGATGCAGA
		R: CGGTCTTCTGGGTCCTCAAT
Mettl3	Human	F: CCCTATGGGACCCTGACAGA
		R: CTGGTTGAAGCCTTGGGGAT
Mettl4	Human	F: ACCAAAATCGCCTCCTCCCAAATC
		R: AGCCACCTCTTTCTCCTCGGAAG
Fto	Human	F: TCAACTGGAAGCACTGTGGAAGAAG
		R: CGAGGCAAGGATGGCAGTCAAG
IGF2BP1	Human	F: ATCGGCAACCTCAACGAGAG
		R: GTTTCGATGGCCTTCATCGC
IGF2BP2	Human	F: CCTGGACACTACCACGTTGA
		R: AACTGATGCCCGCTTAGCTT
IGF2BP3	Human	F: TCACTTCTATGCTTGCCAGGTTGC
		R: CCTTCTGTTGTTGGTGCTGCTTTAC
U6	Human	F: CGCTTCGGCAGCACATATACTAA
		R: AGTGCAGGGTCCGAGGTATT
GAPDH	Human	F: ACGCATTTGGTCGTATTGGG
		R: TGATTTTGGAGGGATCTCGC

**Table 2:** Correlation between CircFAM126A expression and clinicopathological characteristics in PCa

Characteristic	Case (n=46)	CircFAM126A expression		*p*-value
		Low (n=23)	High (n=23)	
Age(year)				0.766
< 65	16	9	7	
≥ 65	30	14	16	
Size (cm)				0.035
< 5	11	9	2	
≥ 5	35	14	21	
Preoperative PSA (ng/ml)				0.373
< 10	26	11	15	
≥ 10	20	12	8	
Microvascular invasion				<0.001
Negative	29	21	8	
Positive	17	2	15	
TNM stage				<0.001
I/II	30	21	9	
III/IV	16	2	14	
Metastasis				0.236
No	25	10	15	
Yes	21	13	8	
